# Integrating complex genomic datasets and tumour cell sensitivity profiles to address a 'simple' question: which patients should get this drug?

**DOI:** 10.1186/1741-7015-7-78

**Published:** 2009-12-14

**Authors:** Cyril Benes, Jeff Settleman

**Affiliations:** 1 Center for Molecular Therapeutics, Massachusetts General Hospital Cancer Center and Harvard Medical School, 149 13th Street, Charlestown, MA 02129, USA

## Abstract

It is becoming increasingly apparent that cancer drug therapies can only reach their full potential through appropriate patient selection. Matching drugs and cancer patients has proven to be a complex challenge, due in large part to the substantial molecular heterogeneity inherent to human cancers. This is not only a major hurdle to the improvement of the use of current treatments but also for the development of novel therapies and the ability to steer them to the relevant clinical indications. In this commentary we discuss recent studies from Kuo *et al*., published this month in *BMC Medicine*, in which they used a panel of cancer cell lines as a model for capturing patient heterogeneity at the genomic and proteomic level in order to identify potential biomarkers for predicting the clinical activity of a novel candidate chemotherapeutic across a patient population. The findings highlight the ability of a 'systems approach' to develop a better understanding of the properties of novel candidate therapeutics and to guide clinical testing and application.

See the associated research paper by Kuo et al: http://www.biomedcentral.com/1741-7015/7/77

## Commentary

The clinical benefit associated with virtually all cancer drugs is typically limited to a fraction of treated patients. Unfortunately, for most of these drugs, the basis for such a variable response to treatment is poorly understood [1]. The recent emergence of so-called 'rationally-targeted' agents, such as the kinase-targeted inhibitors, trastuzumab (anti-HER2 antibody) and the small molecule kinase inhibitors erlotinib (EGFR) and imatinib (BCR-ABL, PDGFR and c-KIT), has led to significant insights into the role of the genomic features of tumour cells in determining the clinical response to these treatments. It has also highlighted the substantial heterogeneity that exists across patient populations with respect to the tumour genome [2-4]. For this class of inhibitors, activating mutations affecting the kinase target have proven to be the most reliable predictors of drug response identified thus far [5-9]. Such findings have prompted substantial efforts to better understand the relationship between specific tumour genotypes and the clinical response to a variety of established and investigational cancer drugs in order to prospectively identify patient cohorts who are most likely to derive clinical benefit from a particular therapeutic [10-14].

However, the identification of 'drug-sensitizing genotypes' for the relatively non-specific conventional chemotherapy drugs has been more challenging. While these agents still constitute the mainstay of first-line cancer drug therapy for many clinical indications, their precise mechanisms of action remain poorly understood which thus challenges efforts to identify the specific genomic determinants of variable treatment response. One approach to this problem has been to interrogate the state of the tumour genome more broadly by exploiting, for example, genome-wide microarray-based expression profiling [15]. Such gene expression profiles, or signatures, can potentially capture complex cellular states that are likely to reflect a mixture of genomic features that vary between tumours and which are associated with both mutational and epigenetic distinctions [16]. Indeed, several such gene signatures, for both predictive and prognostic assessment of patient outcomes, have emerged from pre-clinical as well as clinical studies and a few have now been approved for clinical use [15,17,18]. In addition, a variety of additional forms of systems information, including genomic copy number data, proteomic and phospho-proteomic data, and, more recently, metabolomic information, can all potentially be used to identify distinctions among human tumours that relate to prognosis and treatment response.

In the accompanying report published this month in *BMC Medicine*, Kuo and coworkers present a systems analysis of the sensitivity of a panel of human breast cancer-derived cell lines to a polyamine analogue (PG-11047) currently undergoing early phase clinical testing in cancer [19]. Polyamines are naturally present at relatively high concentrations in all cell types, where they are essential components of nucleic acid metabolism and a variety of fundamental cellular processes [20]. Since the enzymes regulating polyamine synthesis and catabolism are frequently dysregulated in human tumours, they have been considered as potential targets for anti-cancer drug development [21]. The authors had previously established and characterized a collection of breast cancer cell lines as a model system for examining therapeutic efficacy and its relationship to specific genomic features [22]. Although the validity of cell line-based approaches to inform clinical decisions has been the subject of debate for many years, such approaches have recently shown great potential in revealing the genomic basis of anti-cancer drug response [22-26].

Using a panel of 48 genomically characterized human breast cancer cell lines, Kuo *et al*. identified a set of 250 genes whose expression, as assessed by whole genome microarray analysis, was associated with PG-11047 sensitivity in an *in vitro *growth inhibition assay. Then, using a bioinformatics tool called Ingenuity Pathway Analysis, they found that this gene set was enriched for genes associated with interferon response, suggesting that interferon signalling might affect sensitivity to polyamine analogues. This gene set was then further refined through a Monte Carlo cross-validation approach to a list of 13 genes - a manageable number with respect to the evaluation of clinical specimens - and this 13 gene set was found to be predictive of cell line sensitivity to PG-11047. The analysis revealed several findings of potential interest. First, it was observed that cell lines from the basal tumour subtype were more sensitive to PG-11047 than cells from tumours of luminal origin. By applying their classifier to a panel of breast tumour samples, the authors observed that basal tumours were, indeed, predicted to be more sensitive than luminal tumours, suggesting that PG-11047 should potentially be directed to patients with tumours of basal subtype, which is the more aggressive tumour type. Finally, they found that elevated levels of the cellular survival signalling protein, phospho-AKT, were associated with increased PG-11047 sensitivity. Thus, the collective analysis revealed several features of breast tumour cells that may be relevant to their response to PG-11047.

This analysis nicely illustrates how the integration of multiple forms of system wide information with drug sensitivity profiles assessed *in vitro *using cancer-derived cell lines can begin to penetrate the complexity of human tumours. Recent advances in genomic and proteomic technologies [27-29] have led to the establishment of increasingly complex data sets; the use of computational modelling strategies [30] to link such information to drug sensitivity profiles has the potential to substantially enhance our understanding of pharmacologic mechanisms. The ability of the gene signature identified by Kuo *et al*. to facilitate patient selection and to increase the likelihood of a positive clinical outcome remains to be tested. However, this study constitutes a significant step towards the establishment of genomic analysis as a broadly useful strategy for stratifying patients for treatment with agents whose mechanism of action remains poorly understood.

## Competing interests

The authors declare that they have no competing interests.

## Pre-publication history

The pre-publication history for this paper can be accessed here:

http://www.biomedcentral.com/1741-7015/7/78/prepub
